# The Bite of the Honeybee: 2-Heptanone Secreted from Honeybee Mandibles during a Bite Acts as a Local Anaesthetic in Insects and Mammals

**DOI:** 10.1371/journal.pone.0047432

**Published:** 2012-10-16

**Authors:** Alexandros Papachristoforou, Alexia Kagiava, Chrisovalantis Papaefthimiou, Aikaterini Termentzi, Nikolas Fokialakis, Alexios-Leandros Skaltsounis, Max Watkins, Gérard Arnold, George Theophilidis

**Affiliations:** 1 Laboratory of Animal Physiology, School of Biology, Aristotle University of Thessaloniki, Thessaloniki, Greece; 2 Laboratoire Evolution, Génomes, Spéciation, CNRS, Gif-sur-Yvette, France; 3 Université Paris-Sud, Orsay, France; 4 Department of Agricultural Sciences, Biotechnology and Food Science, Cyprus University of Technology, Limassol, Cyprus; 5 The Cyprus Institute of Neurology and Genetics Neuroscience, Nicosia, Cyprus; 6 Department of Pharmacognosy and Natural Products Chemistry, Faculty of Pharmacy, University of Athens, Athens, Greece; 7 Vita (Europe) Limited, Basingstoke, Hampshire, United Kingdom; Alexander Flemming Biomedical Sciences Research Center, Greece

## Abstract

Honeybees secrete 2-heptanone (2-H) from their mandibular glands when they bite. Researchers have identified several possible functions: 2-H could act as an alarm pheromone to recruit guards and soldiers, it could act as a chemical marker, or it could have some other function. The actual role of 2-H in honeybee behaviour remains unresolved. In this study, we show that 2-H acts as an anaesthetic in small arthropods, such as wax moth larva (WML) and Varroa mites, which are paralysed after a honeybee bite. We demonstrated that honeybee mandibles can penetrate the cuticle of WML, introducing less than one nanolitre of 2-H into the WML open circulatory system and causing instantaneous anaesthetization that lasts for a few minutes. The first indication that 2-H acts as a local anaesthetic was that its effect on larval response, inhibition and recovery is very similar to that of lidocaine. We compared the inhibitory effects of 2-H and lidocaine on voltage-gated sodium channels. Although both compounds blocked the hNav1.6 and hNav1.2 channels, lidocaine was slightly more effective, 2.82 times, on hNav.6. In contrast, when the two compounds were tested using an *ex vivo* preparation–the isolated rat sciatic nerve–the function of the two compounds was so similar that we were able to definitively classify 2-H as a local anaesthetic. Using the same method, we showed that 2-H has the fastest inhibitory effect of all alkyl-ketones tested, including the isomers 3- and 4-heptanone. This suggests that natural selection may have favoured 2-H over other, similar compounds because of the associated fitness advantages it confers. Our results reveal a previously unknown role of 2-H in honeybee defensive behaviour and due to its minor neurotoxicity show potential for developing a new local anaesthetic from a natural product, which could be used in human and veterinary medicine.

## Introduction

2-heptanone (2-H) is secreted by the mandibular glands of adult honeybees. The amount of 2-H secreted by an individual bee progressively increases with age, reaching maximum levels in guards and foragers [1]–[3]. The dominant hypothesis about the role of 2-H is that it acts as an alarm pheromone that triggers defensive responses and recruits guards and soldiers in the face of a potential threat [4]–[7]. 2-H could also act as a chemical marker, signalling to other foragers that a flower has already been visited [8], [9]. However, contradictory research results cast doubt on the hypothesis that 2-H acts as an alarm pheromone; it did not trigger any defensive response in honeybees when it was experimentally applied to colonies [2], [10] and it appears to be too volatile to function as a chemical marker on flowers [2], [11]. There is also debate about whether 2-H secreted by guard honeybees attracts or repulses other honeybees; it appears to be attractive in low concentrations and repulsive in higher concentrations [6], [11]. Given all these contradictory results, it is fair to say that the role of 2-H in honeybee behaviour (especially in defensive behaviour) remains unresolved [11].

In an attempt to clarify the role of 2-H, we observed that hive intruders such as wax moth larvae (WML; *Galleria mellonella*) and the parasitic mite, *Varroa destructor*, were paralysed for a short time after being bitten by honeybees. Honeybees use their mandibles to bite invaders that are too small to sting [12], [13]. This led us to suspect that the intruders may have been anaesthetized by 2-H secreted from the mandibular glands during biting. The purpose of this paper is to provide an in-depth investigation of the possible anaesthetic properties of 2-H during honeybee defense and to examine whether 2-H acts as a local anaesthetic on the mammalian peripheral nervous system.

## Results

### Does 2-H Function as an Alarm Pheromone?

**Figure 1:**
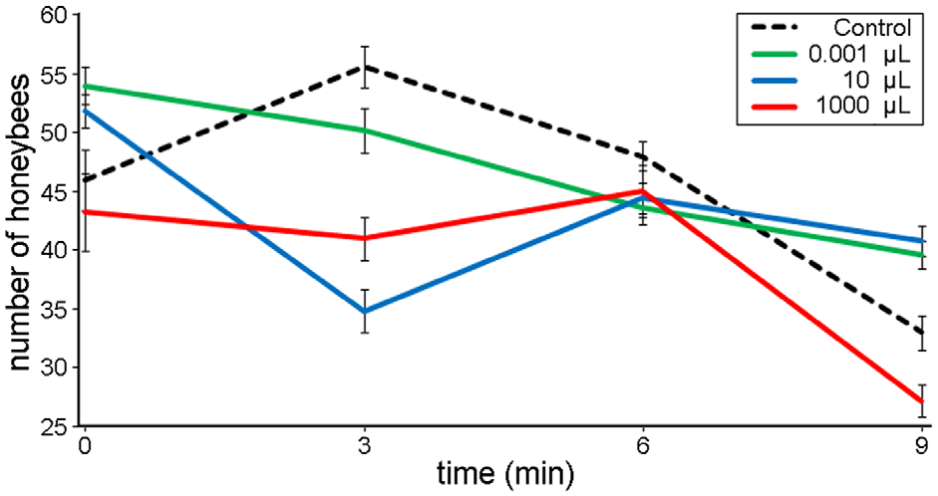
Number of honeybees exiting beehives following 2-H applications . No treatment (control) and doses of 0.001, 10 and 1000 µL of 2-H were applied at the colony entrance. Honeybees were recorded using Electronic BeeSCAN counters.

We first tested the dominant hypothesis that honeybees secrete 2-H as an alarm pheromone that recruits other honeybees in the face of a potential threat. We monitored honeybee response to different doses of 2-H applied at colony entrances. In control colonies, which were not exposed to 2-H, an average of 46.42 bees left the colony per minute (n = 5, SEM = 0.43). There was no statistical difference, 10 minutes after application, between the mean number of bees recruited in control colonies and those recruited in colonies exposed to 0.1 µL 2-H (mean = 47.18, n = 5, SEM = 0.36, p = 1.000). In contrast, higher doses of 2-H acted as a repellent. Compared to controls, significantly fewer honeybees exited the hive per minute in colonies exposed to 10 µL 2-H (mean = 42.54, n = 5, SEM = 0.11, p = 0.003) and to 1000 µL 2-H (mean = 40.88 honeybees, n = 5, SEM = 0.42, p = 0.002) (Fig. 1). The above results clearly show that the application of 2-H did not recruit honeybees from the nest cavity and triggered no defensive responses (no attack on the filter papers containing 2-H).

### Mandible Morphology and 2-H Secretion

**Figure 2:**
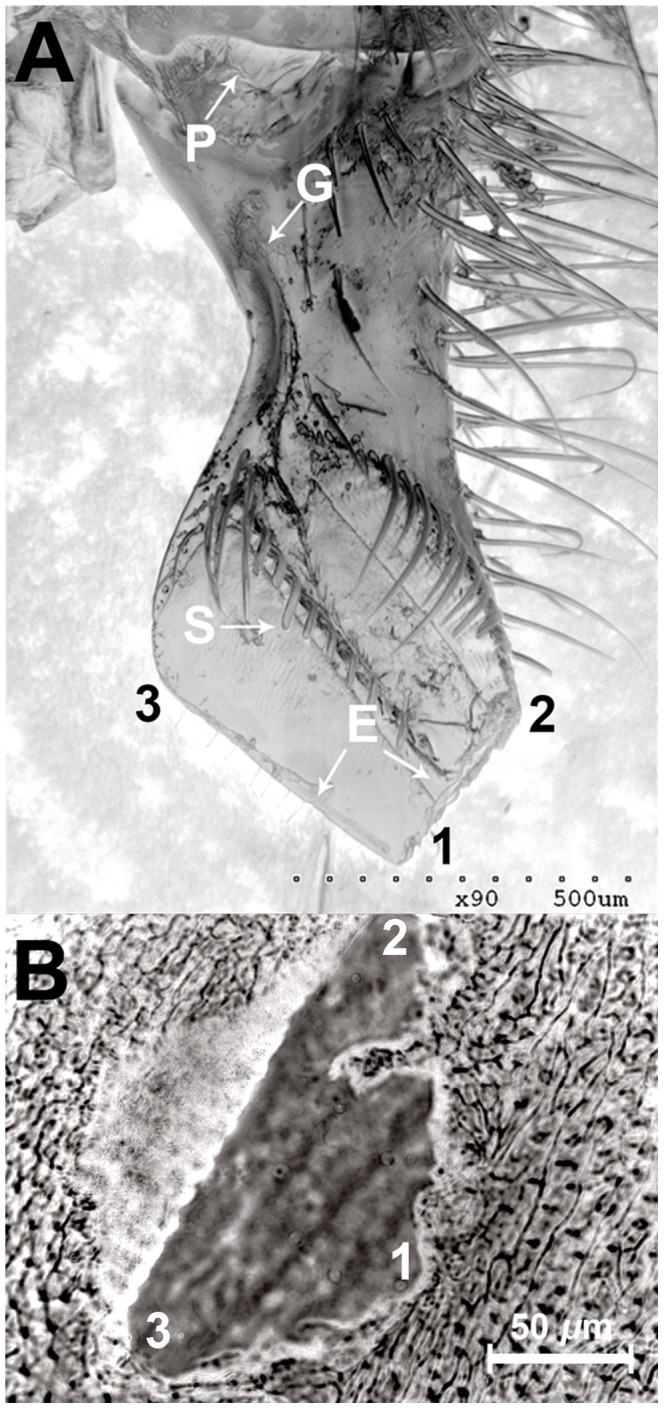
The honeybee mandible and the result of biting wax moth larvae . **a**) SEM scan of a honeybee mandible. P, the pore from which 2-H is secreted; G, groove; S, spikes; E, edges. **b**) the opening created in a wax moth larvae exoskeleton after a honeybee bite. 1, 2, and 3 are parts of the mandible that penetrate the corresponding points on the wax moth larvae exoskeleton.

2-H is secreted from glands on the inner surface of the honeybee mandibles, flows out of a pore (P in Fig. 2a), and is directed through a 440–470 µm long groove (G) in the spatula (S) at the sharp edge of the mandibles. The sharp spatula, assisted by spike-like protrusions on the spatula (S in Fig. 2a), can penetrate the soft cuticle of WML creating a small triangular wound of around 0.01 mm^2^ (Fig. 2b). 2-H enters the intruder’s body cavity through this wound and then dilutes in the plasma of the haemolymph, via the open circulatory system (the solubility of 2-H in water is 4.3 mg/mL).

### Effect of 2-H on Wax Moth Larvae

**Figure 3:**
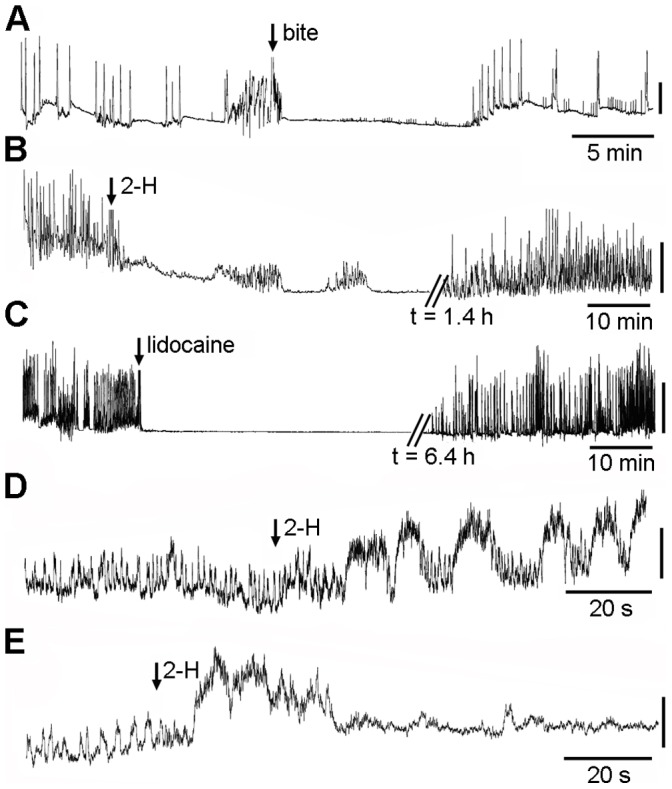
The locomotory pattern of tethered wax moth larvae and the gravitational reflex of *Varroa* mites . **a**) Response of small-size wax moth larvae (WML) bitten by a honeybee, **b**) Response of large-size WML injected with 2.5 µL of 2-H, **c**) response of large-size WML injected with 1.63 mg of lidocaine **d, e)** modification of the gravitation reflex of *Varroa* exposed to 0.025 µL and 0.061 µL of 2-H (respectively), Vertical scale bar: a 3.4 mN, b 9.8 mN, c 75 μΝ, d 9.8 mN.

WML exhibit a constant locomotory pattern that can easily be recorded over an extended period by gently pinning the head to a wax substrate and fixing the tail to the probe of an isometric force displacement transducer [14] (control, Fig. 3a). The locomotory pattern of the small, tethered WML (body weight 0.001–0.002 g) stopped immediately after a honeybee bite, but then gradually recovered within a few minutes (mean = 7.32 min, n = 6, SEM = ±1.51) (Fig. 3a). The locomotory pattern was not affected when we simulated a honeybee bite by applying mechanical pressure to the dorsal region of WML using a pair of fine forceps simulating the pressure caused by the bite.

To identify whether it was, in fact, 2-H that induced paralysis in WML, we performed quantitative and qualitative analyses of guard honeybee mandibular glands using GC-MS and found that each honeybee had an average of 0.0386 µL of 2-H (n = 30, SEM = ±0.0076). Also, we detected an average of 0.00065 µL of 2-H (n = 30, SEM = ±0.00018) in all bitten, paralysed small-sized WML (32.6 µg/1 g of body weight). We detected no 2-H in non-bitten, control WML (n = 10) or in fully-recovered WML (n = 10).

When larger WML (body weight 0.25 g) were injected with an unusually high dose of 2-H (2.5 µL) using a microsyringe (8.15 mg per 1 g body weight), body movement stopped abruptly (Fig. 3b) and recovery was very slow–much slower than recovery observed when the WML was bitten by a honeybee (87.62 min, n = 8, SEM = ±7.21). This suggests that the paralysing effect was dose-dependent. Injection of an equivalent volume of distilled water or physiological saline had no significant effect on the locomotory pattern of the WML, indicating that the paralysing effect was due to 2-H only.

### Effect of 2-H on Varroa Destructor

We tested 2-H on a far smaller arthropod that parasitizes honeybees, the mite, *Varroa destructor*. When 0.025 µL of 2-H was applied topically to the sternum of mites (n = 10) fixed ventral side up, the gravitational reflex–the rhythmic expansion of the thorax and abdomen [15]–was distorted (Fig. 3d). When the concentration was increased to 0.061 µL, the gravitational reflex completely malfunctioned and the *Varroa* mites (n = 10) were totally paralysed within 30–40 s (Fig. 3e) with no recovery, even after 12 h of observation. We applied larger concentrations of 2-H to the *Varroa* mites topically because the mites’ small size made it impossible to inject 2-H into the haemolymph.

### Comparison of Effects of 2-H and Lidocaine on Wax Moth Larvae

The pattern of 2-H action on the WML nervous system–inhibition of the locomotion and recovery (reversible block of excitability)–appears to mimic the pattern of action of local anaesthetics (LAs). To test whether lidocaine, a standard LA [16], has the same pattern of action on WML as 2-H, we injected 1.63 mg of lidocaine (corresponding to 2.5 µL 2-H) into large WML; this dose had a lethal effect in all 12 cases. However, a half dose (0.81 mg) caused a reversible block of the locomotory pattern (Fig. 3c) with full recovery after 6.42 h (n = 5, SEM = ±0.84). These data indicate that 2-H and lidocaine could share a similar pattern of action–a reversible block of locomotion of WML–suggesting that 2-H also acts as an LA and may share a common target with lidocaine. Lidocaine blocks the voltage-gated sodium channels (VGNaCs) of both insects [16] and mammals [17].

### *In vitro* Comparison of Action of 2-H and Lidocaine in Mammalian Cells

**Figure 4:**
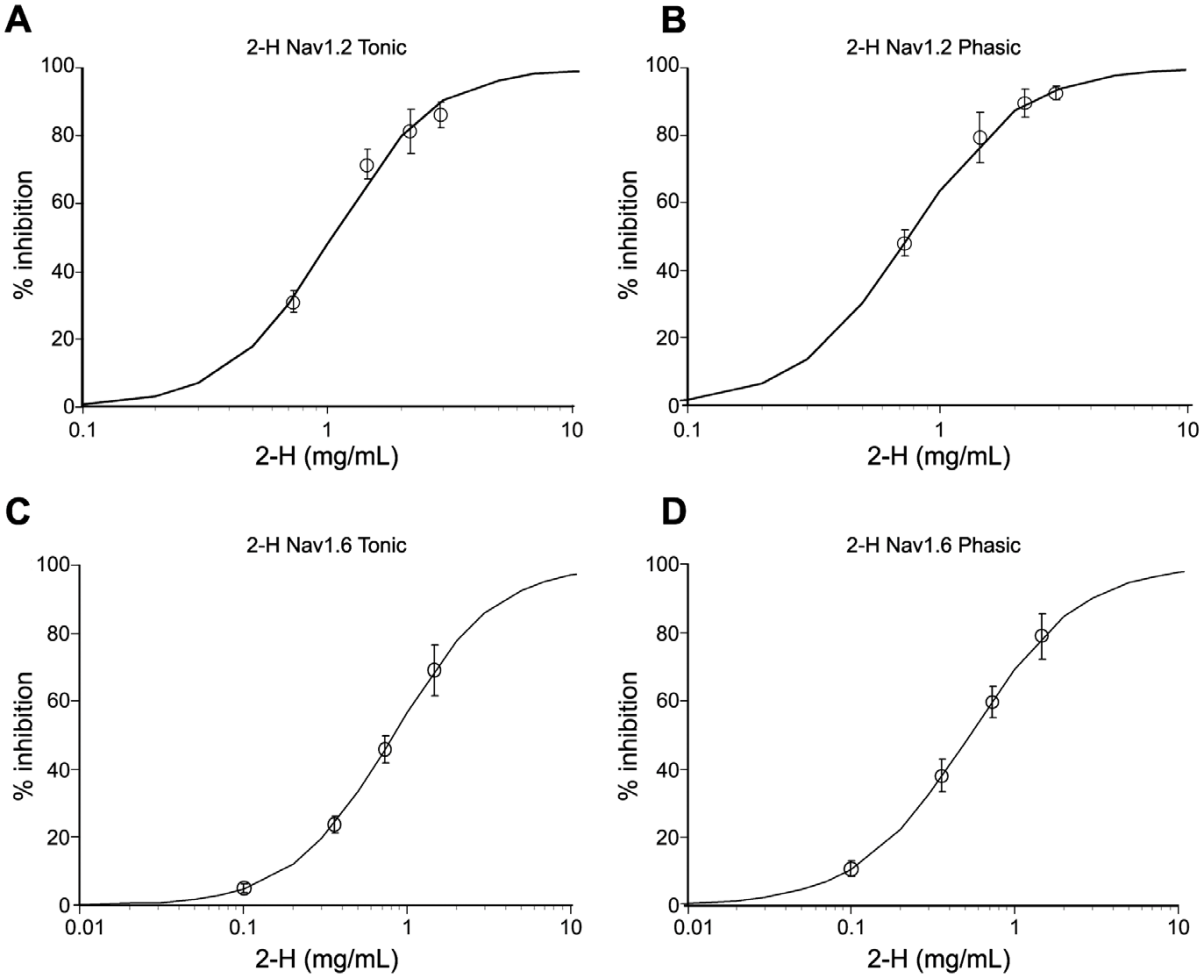
The effect of 2-heptanone on hNav1.2 (a) and hNav1.6 (b) sodium channels . Data represent tonic (a and c) and phasic (b and d) effects. Solid curves represent when data are fitted with a logistic equation with minimum and maximum effects fixed at 0 and 100% (n = 6).

To test whether 2-H can also act by blocking VGNaCs, we used the PatchXpress 7000A to expose mammalian CHO (Chinese hamster ovary) cells transfected with hNav1.2 and hNav1.6 channels to various concentrations of both 2-H and lidocaine. For 2-H, the inward current generated by the depolarization of the cells was recorded within the first 3 min of exposure to eliminate problems created by the high volatility (see Materials and Methods) We plotted the concentration-response curves for 2-H versus hNav1.2 and hNav1.6 channels under tonic and phasic conditions (Fig. 4 a, b, c, d) and estimated the IC_50_ values (concentration required to inhibit function to 50% of normal sodium current) (Table 1). Also, it was possible to estimate the IC_50_ values for lidocaine from similar curves, against the same ionic channels (Table 1). The results shown in Table 1 indicate a discrepancy between the local anaesthetic effect of 2-H and that of lidocaine in the phasic experiments. In the phasic experiments, 2-H was 7.5 times less active than lidocaine for hNav1.2 and hNav1.6. In contrast, in the tonic experiments, 2-H was 3.97 times less active than lidocaine for hNav1.2 and only 2.82 times less active for hNav1.6–a channel located on the nodal region of the nerve fibres [18].

**Table 1:** IC_50_ of 2-H and lidocaine applied to hNav1.2 and hNav1.6 ion channels.

	hNav1.2IC_50_ (mg/mL) (n = 6)		hNav1.6IC_50_ (mg/mL) (n = 6)	
	2-H	lidocaine	2-H	lidocaine
Tonic Experiment	1.03±0.01	0.26±0.01	0.82±0.01	0.29±0.01
Phasic Experiment	0.75±0.09	0.10±0.01	0.53±0.03	0.07±0.01

Values are the mean IC_50_± SEM. *n* = 6 in all cases. 2-H and lidocaine both blocked the two channels that we tested. In the tonic experiments, 2-H was 3.97 (for hNav1.2) and 2.82 (for hNav1.6) times less active than lidocaine. In the phasic experiments, 2-heptanone was 7.50 (for hNav1.2) and 7.57 (for hNav1.6) times less active than lidocaine. The exposure time for 2-H was limited to 3 min.

### *Ex vivo* Comparison of Action of 2-H and Lidocaine on a Mammalian Nerve Fibre

**Figure 5:**
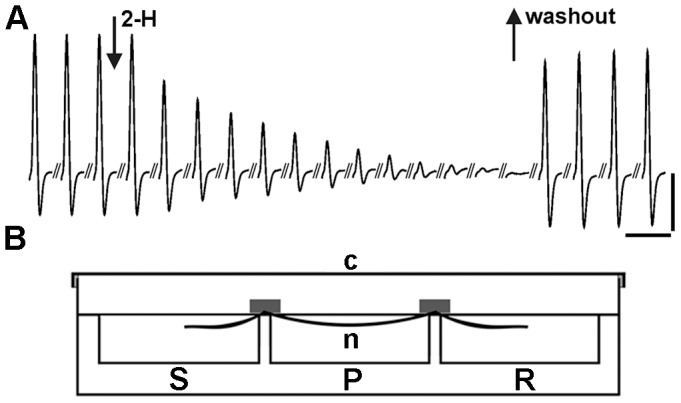
The evoked nCAP from the isolated rat sciatic nerve exposed to saline and 2-H . **a)** The amplitude of the nCAP, baseline to peak, was used as the main parameter to quantify the vitality of the sciatic nerve fibres during exposure to 2-H. The record presents the decrease in the amplitude nCAP during exposure of the sciatic nerve to 3.41 mg/mL 2-H. The first arrow indicates the beginning of the application and the second arrow indicates the time when the nerve was washed and bathed in normal saline. During exposure to 2-H, records were taken at a rate of 1 nCAP per 20s. After 2-H was replaced with normal saline, measurements were taken every 15 min. Vertical scale bar: 3 mV. Horizontal scale bar: 6 ms. **b)**. Diagrammatic representation of the three-chamber recording bath made of Plexiglass. It consists of the recording (R), the perfusion (P) and the stimulating chambers (S), separated by two partitions. The sciatic nerve was placed along the three chambers which were filled with oxygenated saline to cover the nerve. The dimensions of each chamber were 26×26×10 mm (length. width, depth), total volume 10 mL. The cover (c) made of Plexiglass was used to close hermitically the whole recording system, while the air inside the bath was saturated with 2-H, to eliminate the evaporation of 2-H in the perfusion chamber.

Because the *in vitro* experiments indicated that 2-H was 2.82 times less effective than lidocaine for hNav1.6 channels, we used an *ex vivo* preparation–the rat sciatic nerve fibres isolated in a three-chamber recording bath (Fig. 5 b)–to compare the local anaesthetic action of 2-H and lidocaine. The nerve compound action potential (nCAP, Fig. 5a) was used as an index of nerve fibre viability in the recording bath. The advantage of the recording chamber is that the nCAP amplitude remained constant for over 24 h for nerves incubated in saline and stimulated continuously using normal (1 Hz) supramaximal stimulation. After 24 h of continuous stimulation, the amplitude of the nCAP began to gradually decrease due to natural nerve-fibre inactivation [19]. When the nerve fibres were incubated in saline with 3.41 mg/mL 2-H, there was a drastic decrease in the amplitude of the nCAP (Fig. 5, first arrow), and the nCAP was eliminated completely within 220 to 230 s, indicating that all sciatic nerve fibres were inactivated. In this case, the chambers with the saline (the nerves and the recording electrodes) were closed hermetically, using a cover (Fig. 5 b), to prevent evaporation of 2-H. At this stage, the nerves recovered quickly once the sciatic nerve fibres were washed out and the 2-H/saline mixture was replaced with normal saline (Fig. 5 a, second arrow).

**Figure 6:**
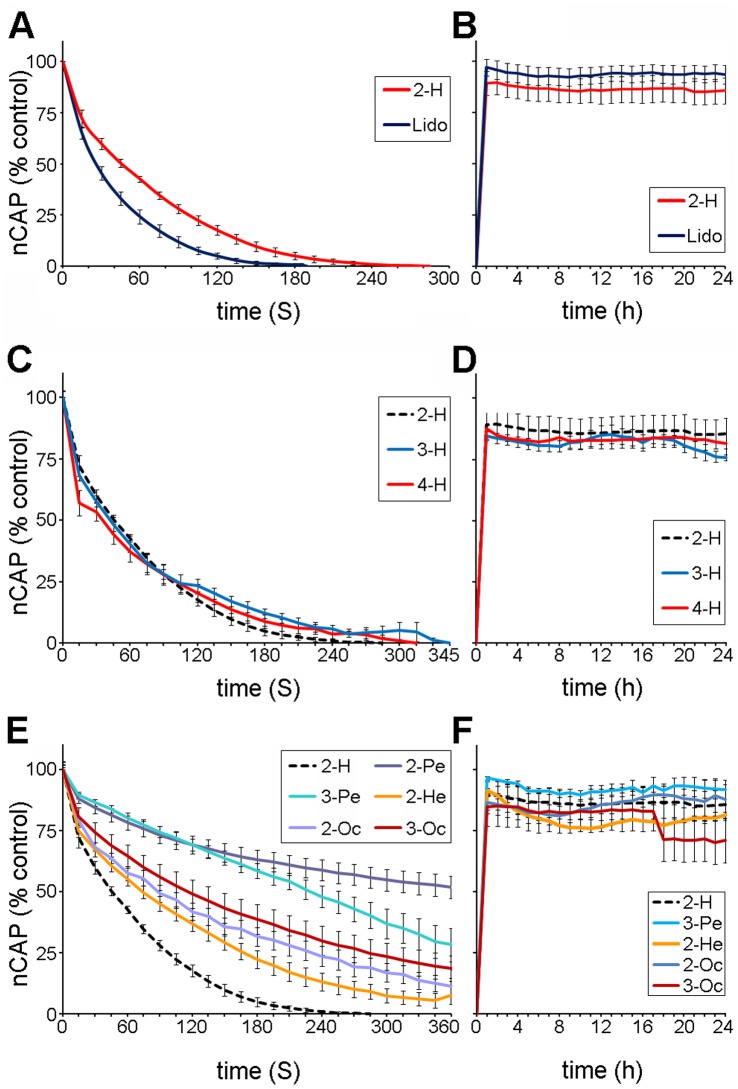
The effect of 2-H, lidocaine, and other alkyl-ketones on the sciatic nerve of the rat . **a)** the effects of 2-H and lidocaine (Lido) on the nCAP of the rat sciatic nerve, the time-response curves for 3.41 mg/mL 2-H and lidocaine. **b**) the time-response curves after the preparation was washed with normal saline (recovery) and the nCAP was monitored for 24 h, **c**) the time-response curves for 3.41 mg/mL 2-H, 3H, 4H, **d**) the recovery **e)** 2-pentanone (2-Pe), 3-pentanone (2-Pe), 2-hexanone (2-He), 2-octanone (2-Oc) and 3-octanone (3-Oc) **f**) the time-response curves after the preparations were washed with normal saline (recovery) and the nCAP was monitored for 24 h.

Using the amplitude of the nCAP, measured from baseline to peak, we plotted the time-response curves (vitality curves) for sciatic nerves exposed to 3.41 mg/mL 2-H and lidocaine under normal stimulating conditions (Fig. 6a). We also plotted the time-response curves for three other concentrations (10.0, 2.28 and 1.41 mg/mL) and used these to estimate the time required for 100% inhibition of the nCAP (called IT_0_) and the time required to inhibit the amplitude of the nCAP to 50% of its control value (called IT_50_) (Table 2). Using the IT_0_ values shown in Table 2 for 2-H and lidocaine, we plotted the concentration-response curves. Thus, the IC_50_ was estimated to be 0.469 (SEM±0.002) for 2-H and 0.337 mg/mL for lidocaine (SEM±0.001).

**Table 2:** The effects of 2-heptanone and lidocaine on the isolated sciatic nerve of the rat.

Substance	Concentration	IT_50_ (seconds)	IT_0_ (seconds)	N
2-heptanone	10 mg/mL	22.3±1.1	195±12	7
	3.41 mg/mL	39.14±4.84	221.88±7.32	12
	2.28 mg/mL	108.9±0.22	358.8±18.07	4
	1.41 mg/mL	109.7±2.87	517.5±25.62	4
IC_50_	0.469 mg/mL (SEM±0.002)			
lidocaine	10.0 mg/mL	12.0±0.95	101.3±2.16	12
	3.41 mg/mL	27.11±3.16	147.50±9.96	12
	2.28 mg/mL	30.9±0.22	186.8±8.07	6
	1.41 mg/mL	32.23±3.16	320.3±14.96	6
IC_50_	0.337 mg/mL (SEM±0.001).			

Furthermore, the effects of 2-H and lidocaine on the sciatic nerve were reversible. The measurements showed that nCAP amplitude recovered to 89.51% (n = 8, SEM±5.87) for 2-H and 95.5% (n = 8, SEM±4.06) for lidocaine when washed with normal saline, and remained at this level for at least 24 h–an indication that neither compound has a significant effect (p>0.05) on the vitality of the sciatic nerve fibres (Fig. 6b).

### Comparison of the Action of 2-H and Similar Compounds on a Mammalian Nerve Fibre

We also plotted the time-response curves for 3.41 mg/mL of the heptanone isomers, 3-heptanone (3-H) and 4-heptanone (4-H) (Fig. 6 c and d), as well as of other alkyl-ketones (Fig. 6e and f). Although the IT_50_ for 2-H was not significantly different from 3-H and 4-H (Table 3), 2-H is more active overall–the IT_0_ for 2-H was significantly lower (p<0.05) than that of 3-H and 4-H (Table 3). 2-H had the fastest inhibitory effect on the nCAP of all of the remaining ketones examined. The IT_50_ for 2-H was significantly different from that of 2-hexanone, 2-octanone, 3-octanone, 2-pentanone and 3-pentanone (Table 3). Also, the IT_0_ for 2-H was significantly lower (221.88 s) than all of the other ketones (from 400 to 765 s for all but 2-pentanone, which was greater than 24 h; Table 3).

**Table 3:** Comparing the local anaesthetic properties of 2-heptanone with the other ketones.

Substance3.41 mg/mL	IT_50_	IT_0_	n
2-heptanone	39.14±4.84	221.88±7.32^a^	12
3-heptanone	40.82±1.67	258.00±11.47^b^	6
4-heptanone	45.24±0.44	283.57±16.89^b^	8
2-hexanone	74.42±0.79	401.00±33.20	7
2-octanone	81.45±1.58	555.00±75.00	4
3-octanone	117.10±0.53	722.50±95.66	6
2-pentanone	390.60±0.10	No measurable	6
3-pentanone	230.30±1.29	765.00±86.60	4

a,b Significant difference (p<0.05) for the IT_0_ comparison between 2-heptanone, 3-heptanone and 4-heptanone.

2-H, the other heptanones (3-H and 4-H), and the rest of the alkyl-ketones that we examined reversibly blocked the excitability of the rat sciatic nerve. Recovery of the nCAP, after the saline + compound was removed and replaced with normal saline, was monitored for 24 h and was almost identical for all compounds–80 to 95% of the control (Fig. 6 b, d and f).

## Discussion

We used field tests to investigate the current dominant hypothesis that 2-H, secreted from glands in honeybee mandibles, functions as an alarm pheromone that stimulates defensive behavioural reactions against a potential threat. The results recorded by electronic counters at the hive entrance clearly showed no recruitment of defensive honeybees from the nest cavity, supporting previous results obtained using different methods [2], [10]. Guard honeybee behaviour was normal at low doses of 2-H and we noted no other defensive responses, such as stinging behaviour. If 2-H were acting as an alarm pheromone, the responses should be instantaneous–electroantenogram recordings have shown that honeybees can easily detect the presence of 2-H [20]. Though 2-H is highly volatile and easily detectable by honeybees at low concentrations (4.049 µg/mL; molar fraction = 6.19), the absence of any reaction during our research or during experiments where 2-H was delivered to the hive entrance via air pumping [10] suggests that it is unlikely that 2-H functions in pheromonal “emission-reception” transmission from a guard honeybee to the colony.

The present study revealed that in addition to injecting venom via a sting, honeybees can use 2-H for defence, to paralyse invaders that are too small to sting. 2-H is produced in the mandibular glands, released by the mandible pore of a reservoir and through the groove flows at the sharp edge of mandibles. We believe, based on our morphological studies and the anatomical evidence provided by others [21], that the release of 2-H is not passive, but actively controlled by the contraction of mandibular muscles. Honeybees continuously use their mandibles for many tasks during their life cycle, and it is obviously not practical to release 2-H with every bite. We assume that 2-H is released only during a strong defensive bite, since the reservoir of 2-H is closely associated with other mandibular muscles and is very close to the apodeme of the adductor muscle, running along the reservoir in a parallel position [21]. Thus, powerful contraction of these muscles during a strong defensive bite may put pressure on the reservoir, releasing 2-H out of the pore to the edge of the sharp spatula. We have shown that during such a bite, the spatula penetrates the cuticle of a parasite, such as WML, to inject 2-H into the plasma of the haemolymph. The open circulatory system of the parasite allows for instant perfusion of 2-H to almost all parts of the body. Our results have shown that 0.00065 µL/WML of 2-H is sufficient to paralyse the invaders, but only for a few minutes–enough time for them to be removed from the beehive. This defensive tactic–injecting “venom” through the mandibles during a strong bite–appears to parallel that of other species such as snakes, which inject venom through their teeth during a strong (i.e. defensive) bite.

The rapid recovery of the WML that were injected with 2-H, combined with the fact that 2-H was never found in recovered specimens during our GC-MS studies, indicated that this compound is metabolised quickly, possibly through the insect cytochrome P450 system [22]. Although there is no data for the metabolism of 2-H in insects, in male Fischer 344 rats, 2-H is metabolised to CO_2_, acetate and a variety of compounds that could be either anabolic or catabolic or a combination of the two [23]. Although this is still a hypothesis, WML recovery after injection with lidocaine suggested that WML have the potential to metabolise this LA, probably by insect cytochrome P450. Lidocaine is metabolized by human hepatic microsomes and purified human cytochrome P450s to monoethylglycinexylidide (MEGX) and 3-hydroxylidocaine (3-OH-LID) [24].

The pattern of 2-H action on WML is a fast, dose-dependent inhibition of locomotory pattern followed by a fast recovery. In invertebrates and vertebrates, locomotion is generally produced by a central pattern generator (CPG)–a neural network that consists of interneurons and motoneurons interacting via synaptic terminals. Also, the CPG is driven by sensory neurons and their axons, while it drives the muscles through motor axons and neuromuscular junctions [25]. The instant paralysis of WML caused by 2-H and the fast recovery indicates that this chain of neuronal control was interrupted at one or more points, but only for a few minutes. In the case of another invertebrate, the mite *Varroa destructor,* this path interruption is irreversible and leads to death, probably due to this ectoparasite’s small size. Honeybees use the so-called “grooming” behaviour to remove mites from the bodies of their nest mates; they use their mandibles to bite and remove *Varroa* [13], [26], [27]. Colonies in which high numbers of dead and damaged mites accumulate at the beehive bottom board are correlated with effective hygienic behaviour through grooming. It is possible that this ability is facilitated by fast and effective biting of mites, depositing 2-H on the cuticle of the *Varroa*, which in turn causes paralysis and death.

Fast inhibition of neural activity followed by fast recovery is an effect typical of local anaesthetics. The fact that lidocaine, the standard local anaesthetic, was found to have a similar pattern to that of 2-H on WML in our experiment, combined with the fact that lidocaine acts on insect and mammalian VGNaCs [16], was the first indication that 2-H may act as a local anaesthetic on the nervous system of WML. This finding led us to compare the action of 2-H with that of lidocaine.

We tested and compared the effects of 2-H and lidocaine *in vitro* on hNav1.2 and hNav1.6 channels and *ex vivo* on the rat sciatic nerve fibres. *In vitro,* the patch-clamp studies showed a discrepancy; in the phasic experiments 2-H was 7.5 times less effective than lidocaine. However, in the tonic tests on hNav1.6, 2H was 2.82 times less effective than lidocaine. This similarity between 2-H and lidocaine, may also indicate a local anaesthetic action of 2-H, since the hNav1.6 channel is located in the nodal area of the peripheral nerve fibres [18] and is obviously involved in inhibition of the action potential caused by local anaesthetics [28].

In the *ex vivo* studies, 3.41 mg/mL of lidocaine eliminated the nCAP in 147.55 s while the same concentration of 2-H took 221.88 s–a delay of only 70–75 s, a relatively short period given that local anaesthesia can last for over 2 h. There was no significant different in recovery after exposure to 2-H or lidocaine (89.5 and 95.5% recover within the same time: 30–45 min). Furthermore the IC_50_ was 1.4 times higher for 2-H than lidocaine (2-H was 1.4 times less effective). Thus, comparison of the two compounds using the isolated sciatic nerve preparation–used extensively to assess local anaesthetic properties of other compounds [29]–[34]–has shown that 2-H and lidocaine have a similar action, further supporting the classification of 2-H as a compound with local anaesthetic properties.

The results of the *in vitro* and *ex vivo* experiments were quite similar (IC_50_ of 2-H 2.8 *VS* 1.4 times higher than lidocaine), in spite of the fact that the patch-clamp tests applied only to a single ion channel while the experiments on the nerve fibre applied to all ion channels involved in generating the action potential. Though there is a difference, it is important to bear in mind that interpretation of the experiments on whole nerve fibres, where the action potential is an interaction between the activation of many Nav channels, is more complex. For example, the Nav1.6 channel is expressed alone or together with Nav1.1 in the nodes of Ranvier [18] while Nav1.8 and Nav1.7 are expressed in the sensory neurons [28] of the sciatic nerve fibres. Furthermore, it is possible that there is a species difference (hamster-rat) between the *in vitro* and the *ex vivo* methods there is a species difference that could very well explain the observed differences; species differences have been found for other compounds that affect VGNaCs [35]. Thus, we believe that the results obtained using the whole-sciatic nerve preparation, which indicate a local anaesthetic action of 2-H, could be equally reliable to those of the single ion channel experiment. Therefore, 2-H, with an IC_50_ just 1.4 times higher than that of lidocaine, can also be classified as a local anaesthetic.

At this point, it is worth mentioning that 2-H had the fastest inhibitory effect when compared to 3-H and 4-H, with an IT_0_ of 221s. Heptanones were generally found to be far more effective than all the other alkyl-ketones examined, such as 2,3-pentanones, 2-hexanone, and 2,3-octanones. The fact that 2-H has the fastest response of all the other alkyl-ketones may answer the question, “Why do honeybees inject 2-H and not another ketone?” We assume that natural selection favoured 2-H due to the associated fitness advantages it confers over other, similar compounds (fastest inhibitory effect on the nervous system). Furthermore, it seems that 2-H was also “selected” for other reasons, since one of its main advantages is that its IC_50_, assessed both *in vitro* and *ex vivo*, is far below its solubility in water. A convincing example is the fact that 3.41 mg/mL eliminated the nCAP of the sciatic nerve within 221 s while a concentration of 10 mg/mL, about three times higher, eliminates the nCAP in only slightly less time–195 s.

Animal venoms are often used to create a range of medicines [36], [37]; this study found that 2-H has an equivalent action to lidocaine when examined using the whole-nerve preparation. The question here is whether 2-H can pass the preclinical and clinical tests required in order to be considered an LA that can be used in clinical practice. There is evidence in favour of exploring 2-H as a new LA. The 24 h monitoring of nerve vitality showed no neurotoxic action of 2-H or lidocaine within the time limits of the particular *ex vivo* method. Also, 2-H has been found to have no neurotoxic effects when tested *in vivo* on the nerves of the rat tail [38], while lidocaine has been associated with some neurotoxic effects [32], [39]–[41]. For example, it has been shown that 80 mM lidocaine disrupts the axonal membrane of the rat sciatic nerve *in vitro* [32]. Given that lidocaine is used extensively in clinical practice at concentrations of 2% (or 20 mg/mL 80 mM), there is a real motivation to develop new and possibly less toxic local anaesthetics. 2-H could be a good candidate since a) it is a natural product (Cone snail venoms have been used already as analgesics [42]), b) it presents excellent local anaesthetic properties, equivalent to lidocaine, c) it has a lipophilicity near that of lidocaine (logP for 2-H is 1.98 and for lidocaine is 2.26), d) it has a maximum effectiveness below its solubility in water, e) it is metabolised over the same time frame as lidocaine, at least in invertebrates. Furthermore, 2-H is generally less toxic, with an acute LD_50_ of 1670 mg/kg (oral rat) compared to 313 mg/kg for lidocaine. The FDA has evaluated the safety profile for 2-H and describes the substance as a “food additive permitted for direct addition to food for human consumption" (21 CFR 172.515). The discovery of a new local anaesthetic derived from honeybees opens exciting new opportunities both for research into honeybee defensive behaviour and for pharmaceutical research into the development of a new, potentially less toxic, local anaesthetic.

## Materials and Methods

### Ethics Statement

Samples of honeybees and wax moth larvae were obtained from A. Papachristoforou’s private apiary or with permission from the private apiary of Argiris Grigoriou (city of Larnaca), located in the Republic of Cyprus. No specific permits were required for the described field studies (involving honeybee colonies) since they were conducted on AP’s private apiary. All experimental procedures on the rat complied with the protocols outlined by the ethics committee of Aristotle University of Thessaloniki, Greece, regarding the recommended standard practices for Biological Investigations (License Number: 30497). Also, the experiments were approved by the local Veterinary Authorities (Ministry of Agricultural Development and Food, Thessaloniki 09/06/2011, Protocol Number 144256/864).

### Monitoring the Recruitment of Defensive Honeybees with BEESCAN Counters

We conducted field experiments at the apiary, in which we used electronic monitoring devices [43] (BeeSCAN v2.03) to test whether the presence of 2-H on the colony flight boards stimulated recruitment of defensive honeybees. The monitoring devices counted the number of honeybees exiting the beehive once per minute for 10 minutes for each of the five colonies per treatment. An increase in the number of bees leaving the colony compared to controls indicates that more bees are being recruited to the flight board to participate in defence. A decrease in the number of bees leaving the colony compared to controls indicates that bees are being repelled from the flight boards. To apply 2-H, we followed the standard methodology described in previous experiments [2].

### Recording the Locomotory Pattern of Wax Moth Larvae

To monitor the constant locomotory pattern of WML, the probe of a force-displacement transducer (FT-03C, Grass Instrument Company, USA), attached to a micromanipulator, was hooked onto the tail of the WML, while the head was carefully pinned to a wax substrate [44], [14]. The electrical signal generated by the rhythmic force produced during locomotion was amplified and fed to a computer through a data acquisition card interface (Keithley KPCI 3102, Keithley Instruments, Cleveland, OH, USA). Software designed using the facilities of Labview (Labview 5.1, National Instruments USA) permitted analysis of the recorded data.

### Patch-clamp Tests

To test whether 2-H can act by blocking VGNaCs, the PatchXpress 7000A was used. Mammalian CHO (Chinese hamster ovary) transfected with ion hNav1.2 and hNav1.6 channels cDNAs (SCN2A and SCN8A genes) were exposed to 2-H under both tonic and phasic conditions. The mammalian CHO-K1 epithelial embryonic cell line (ATCC number CCL-61) from *Cricetulus griseus* was obtained from ATCC, Manassas, VA. CHO-K1 cells were transfected with either hNav1.2 or hNav1.6 channel cDNA (SCN2A and SCN8A genes, respectively) and were exposed to 2-H under both tonic and phasic conditions.

The effects of 2-H were evaluated on hNav1.2 at 0.73, 1.46, 2.19, and 2.92 mg/mL and on hNav1.6 channels at 0.1, 0.36, 0.73, and 1.46 mg/mL, with each concentration tested in at least six cells (n = 6). Cells were exposed to each concentration of 2-H and measurements of the inward current were obtained within the first 3 min of exposure. This was necessary due to the high volatility of 2-H. The records showed that hNav1.2 and hNav1.6 channels gradually recovered from the inhibitory effect of 2-H after 4–6 min of exposure, due to 2-H evaporating from the saline solution. Since the PatchXpress has open cell chambers where the testing occurs, there is no way to hermetically seal the experiment.

For comparison, the action of lidocaine was also tested on hNav1.2 and hNav1.6 channels at concentrations of 0.0270, 0.2030, 0.4060 and 1.0832 mg/mL. All experiments were performed at room temperature. The effects of 2-H and lidocaine on the tonic and phasic block of sodium current were also quantified by fitting concentration–response curves using non-linear regression techniques (Hill equation, minimum and maximum effects fixed at 0 and 100 [n = 6]). Based on this analysis, we estimated the IC_50_.

### GC-MS Analysis

#### Extraction procedure

We measured the 2-H content of the following types of samples: a) isolated honeybee mandibular glands (n = 30); b) wax moth larvae (WML; body weight of around 0.01 g) that had been bitten by honeybees (n = 30) –2-H extracted immediately after paralysis; c) WML that had been bitten by honeybees (n = 10) –2-H extracted after full recovery; d) non-bitten WML (controls, n = 10).

To optimize the detection of 2-H, all samples were 1) treated with liquid nitrogen in order to stop any enzymatic process and ensure complete lyses of cell walls and then 2) extracted with ethanol. The extracts were centrifuged, filtered, and stored at −20°C until analysis.

All honeybees used in this study were guards from *Apis mellifera cypria*, *Apis mellifera ligustica* and *Apis mellifera macedonica* colonies. We determined which bees were guards through visual observation of colony entrances; guard bees were defined as those that were observed patrolling the entrance and inspecting incoming honeybees. When bees were required to bite, we used forceps to place them in close proximity to moving wax moth larvae and then applied light pressure to their thoraxes to induce them to bite.

#### GC-MS conditions

Chromatographic analyses were carried out on a Hewlett Packard 6890 gas chromatographic model hyphenated to a Hewlett Packard 5973 mass selective detector using electron impact (70 eV) as the ionization source, further equipped with an Agilent 6890 autosampler. An HP 5MS (30 m×0.25 mm; film thickness 0.25 µm) column was used for the separations. Helium was used as the carrier gas (flow 0.8 ml/min). The oven temperature was programmed from 40 to 60°C at a programming rate of 2°C min^−1^ and then raised by 10°C min^−1^ increments from 60 to 280°C (8 min hold). The temperatures of the injector and transfer line were set at 220°C and 280°C, respectively. Samples were injected directly with a sampling volume of 2 µL; the analyses were performed in the split mode (1∶5). 2-H was identified by comparing its retention time (Rt) and mass spectra with those of authentic samples (Sigma-Aldrich), as well as from the Wiley library spectra match. Thus, under the above chromatographic conditions, the Rt of 2-H was 8.47 min and the ions monitored at EI (70eV, full scan) were m/z 43, 58, 71, 85, 99 and 114. Quantification, however, was performed at Selected Ion Monitoring (SIM) mode and the ions were monitored at m/z 58 and 71.

#### Calibration curve

To generate the calibration curve, we prepared 12 dilutions of 2-H (obtained from Sigma-Aldrich) with concentrations ranging between 0.0001 and 0.075 µL/mL. The calibration curve was obtained by plotting the peak areas against the analyte concentrations. A generalized reduced gradient algorithm was applied using Excel Solver 2003 in order to identify the appropriate linear calibration curve. The calibration curve used eliminates the sum of the total deviation for 2-H concentrations subjected to specific constraints that didn’t allow any concentration to exceed more than 15% of the initial experimental values. The solution provided the following equation: Y = 10665.94*X*10^4^+12035.43 , where Y is the peak area that is derived from the chromatogram and X is the concentration of 2-H. We then conducted further sensitivity analyses, in which the limits report indicated that the range for the two coefficients were *(10665.94, 10686.50)* and *(12035.43, 12675.95)*, respectively. These values did not violate any of the predefined constraints.

We evaluated the method’s selectivity by injecting six control extracts, prepared using the described extraction procedure. At the limit of quantification (LOQ), we did not observe any interference at the retention time corresponding to 2-H, demonstrating that the methodology has adequate specificity.

The method’s accuracy (mean relative error [% bias] between the nominal and measured concentrations) was adequate, and was within±15% for each concentration level.

Five concentration levels (3 samples for each), 0.0001, 0.0005, 0.001, 0.01, and 0.075 µL/mL of 2-H, were used to estimate intra- and inter-day precision–measured as the relative standard deviation (RSD) of replicate assay–and accuracy–measured as relative error RE ([nominal concentration-measured concentration]/nominal concentration)x100. For the inter-day calculations, the samples were injected on 5 different days. The RSDs were below 10% for both intra- and inter-day precision measurements. The REs varied between −15% and 9% for both intra- and inter-day accuracy. All accuracy and precision data are presented in Table S1.

The sensitivity of the method was evaluated using the values of limit of detection (LOD) and limit of quantification (LOQ). LOD determines the lowest amount of 2-H that can be detected using this procedure and was calculated as the lowest concentrations tested that yield a signal-to-noise ratio (S/N) of at least 3. LOQ reflects the lowest concentration of the compound that can be measured and refers to a signal-to-noise ratio of 10. For this purpose, ten different blank ethanol samples were injected. LOD was estimated at 5×10^−7^ and LOQ at 5×10^−6^ µL/mL 2-H.

### The *ex vivo* Rat Sciatic Nerve Preparation

The *ex vivo* studies used both male and female rats weighing between 230 and 250 g. Animals were sacrificed using light anaesthesia with CO_2_ and cervical dislocation. The sciatic nerve of the rat, consisting of 27,000 sciatic nerve fibres [45], was isolated in a three-chamber recording bath (Fig. 5b). The sciatic nerves were dissected from the spinal cord to the knee, immersed in oxygenated (O_2_, 100%) saline solution and cleaned. The composition of the saline was (in mmoles/L): 136 NaCl, 11 glucose, 4.7 KCl, 2.4 CaCl_2_, 1.1 MgCl_2_, 1 NaHCO_3_, 10 HEPES, pH = 7.2). All experiments were performed at a constant temperature, 26.0±1.0°C. The epineurium and the perineurium were removed under a dissection stereoscope using fine forceps and micropins to allow better access of the drugs to the nerve fibres. The nerve was mounted in a three-chambered recording bath made of Plexiglas, which is described elsewhere [29], [30]. The recording bath consisted of the recording (R in Fig. 5b), perfusion (P) and stimulating (S) chambers, each with a volume of 12 mL. The nCAP (Fig. 5a) was generated by stimulating (1 Hz) the part of the nerve in the stimulating chamber and was recorded in the recording chamber every second using standard electrophysiological methods. The nCAP was monitored throughout the experiment as an indication of the vitality of the nerve fibres in the recording bath. To expose the nerve to the compound under investigation (2-H, lidocaine) the compound was diluted in oxygenated saline to the desired concentration, and minor changes in pH were corrected. The concentrations of 2-H and lidocaine tested are given in Table 2. Then, the 12.0 mL saline in the perfusion chamber was replaced with saline diluted with either 2-H or lidocaine. The local anaesthetic effect began with a fast decrease of the nCAP. Immediately after the nCAP was eliminated, the saline+compound was removed and the nerve was washed out. Then, recovery of the nCAP in normal stagnant saline was monitored for 24 h. It is worth noting that the recording bath is designed in such a way that the chambers with the saline and 2-H (the nerves and the recording electrodes) was shielded from the air by using the cover (c in Fig. 5b) to avoid evaporation of the volatile 2-H. The saline with 2-H was added through a small diameter opening on the cover.

### Data Analysis-statistics

For further data analysis, the amplitude of the nCAPs, estimated from baseline to peak (Fig. 5), were measured at a specific time (every 1.0 s, 1.0 min or 1.0 h) and were expressed as a percentage of the amplitude of the nCAP at t = 0 (which was considered to be 100%). In the neuropharmacological experiments, time t = 0 was considered the time before application of the compound under investigation. The time-response curves (or vitality curves) for a specific concentration of 2-H and lidocaine were plotted using values derived from the replicate experiments (n) that were averaged and expressed as a mean±standard error of the mean (SEM). The concentrations tested are given in Table 1. From the time-response curves generated using the program GraphPad Prism 5.0, the IT_50_ and IT_100_ were estimated for a specific concentration of 2-H and lidocaine. Using the IT_50_ values, it was possible to estimate the IC_50_ for 2-H and lidocaine. The data were fitted in the program GraphPad Prism 5.0 using a one-phase exponential decay equation for the IC_50_. Data were analyzed by one-way ANOVA and Bonferroni’s post hoc tests, when required. Significance was determined using Students t-test or ANOVA as appropriate (p<0.05).

### Chemicals

KCl, NaCl and MgCl2 were from Panreac (Spain), HEPES from Biochemica Fluka (Switzerland), glucose from Riedel-de Haen (Germany) and NaHCO3 from Merck (Germany). Lidocaine was purchased from Sigma (Deisenhofen, Germany), 2-heptanone from Sigma–Aldrich (Germany).

## Supporting Information

Table S1 **Intra- and inter-day precision and accuracy data for 2-H calculations.**
(DOC)
