# Supplementary material for: The Bite of the Honeybee: 2-Heptanone Secreted from Honeybee Mandibles during a Bite Acts as a Local Anaesthetic in Insects and Mammals
Source: PLoS One. 2012 Oct 16;7(10):e47432. doi: 10.1371/journal.pone.0047432 (PMC3472974; doi:10.1371/journal.pone.0047432)
Supplement: Table S1 — Intra- and inter-day precision and accuracy data for 2-H calculations. (DOC) [file pone.0047432.s001.doc]

|  | Nominal concentrations (μL/mL*10^4^) | Measured concentrations (μL/mL*10^4^±SD) | Relative standard deviation % | Relative error % |
| --- | --- | --- | --- | --- |
| Intra-day | 1 | 0.91±0.09 | 9.73 | 8.99 |
|  | 5 | 4.80±0.15 | 3.02 | 4.09 |
|  | 10 | 11.43±0.74 | 6.50 | -14.26 |
|  | 100 | 104.00±3.49 | 3.35 | -4.00 |
|  | 750 | 750.00±22.34 | 2.98 | 0.01 |
| Inter-day | 1 | 1.05±0.07 | 6.32 | -4.46 |
|  | 5 | 4.59±0.42 | 9.19 | 8.12 |
|  | 10 | 9.48±0.67 | 7.05 | 5.25 |
|  | 100 | 102.92±6.93 | 6.73 | -2.92 |
|  | 750 | 766.92±36.58 | 4.77 | -2.26 |
